# Metagenome-assembled genome of *Pseudomonas* sp. from coenocytic alga *Vaucheria bursata*

**DOI:** 10.1128/mra.00485-24

**Published:** 2024-10-14

**Authors:** Gerardo Laureano, Abdiel Esparra Bermudez, Ramón E. Rivera Vicéns, Alok Arun

**Affiliations:** 1 Institute of Sustainable Biotechnology, Inter American University of Puerto Rico, Barranquitas, Puerto Rico, USA; 2 Department of Biological Sciences, California State University Stanislaus, Turlock, California, USA; The University of Arizona, Tucson, Arizona, USA

**Keywords:** genomes, cyanobacteria, algae

## Abstract

Here, we report a draft metagenome-assembled genome and annotation of *Pseudomonas* sp. obtained from the sequenced genome of *Vaucheria bursata*. The genome completeness was 97.9% with a total of 5,322 open reading frames, of which 3,147 genes were annotated. The availability of the metagenome will elucidate the possible epiphytic interactions of bacteria with *Vaucheria bursata*.

## ANNOUNCEMENT

The genus *Pseudomonas* is a Gram-negative bacterium from the class Gammaproteobacteria. These bacteria show great metabolic adaptability and can colonize a wide range of natural habitats and adopt various lifestyles (1, 2). Some species of *Pseudomonas* exhibit epiphytic relationships as observed in the marine alga *Saccharina latissimi* (3). In our current study, a metagenome-assembled genome (MAG) of *Pseudomonas* sp. was obtained and assembled from the genomic DNA sequencing data of the freshwater alga *Vaucheria bursata*. Our study supports an earlier finding suggesting the presence of endophytic bacteria in *Vaucheria* sp. (4).

Axenic cultures of *Vaucheria bursata* were obtained from the UTEX culture collection of algae at UT-Austin (Austin, USA). Vegetative filaments were grown in 3N Bold Modified medium under a photo cycle of 16L:8D at 25°C. Total genomic DNA was extracted from filaments using the commercial kit NucleoSpin Plant II from Macherey-Nagel according to the manufacturer’s instructions. The libraries were prepared using Illumina’s Nextera DNA Flex Library Preparation Kit as per the manufacturer’s protocol. A standard 350-base pair (bp) DNA library was prepared, and the Illumina NovaSeq sequencing platform was used to sequence the genome using paired-end reads (2 × 150 bp). A total of 167,750,552 reads provided a sequencing coverage of 90×. All software was used in their default parameter unless otherwise specified. Low-quality reads and quality verification were performed using Fastp version 0.23.4 (4). MetaWrap version 1.3 pipeline was applied to construct the MAG (5). The draft MAG showed strong similarity to *Pseudomonas,* CheckM completeness, and contamination of 97.9% and 0.8%. ANI analysis from MiGA showed a completeness of 95.3% and a quality score of 90.0% (6). BUSCO analysis was also performed using the lineage Pseudomonadales resulting in a genome completeness of 98.8% (i.e., single copy = 98.7%, duplicates = 0.1%) (7).

The constructed *Pseudomonas* sp. MAG showed a total genome length of 5,795,005 bp (*N*50 = 141,278 bp), distributed across 61 contigs, with a GC content of 60.13% (Table 1). Gene prediction and annotation were performed using Prokka in the European Galaxy platform (8). A total of 5,322 open reading frames (ORFs) were predicted, of which 3,147 were annotated and the remaining were hypothetical proteins.

**TABLE 1:** Assembly and annotation features of the MAG *Pseudomonas* spp.

Parameters	Statistics
Estimated genome size (Mb)	5.79
Number of contigs	61
*N*50 (bp)	141,278
GC (%)	60.13
Number of ORFs	5,322
Annotated genes	3,147

A further genome analysis resulted in the identification of two genes namely photosystem I assembly protein Ycf3 and photosynthetic apparatus regulatory protein RegA with possible roles in photosynthesis. It has been reported that Ycf3 is associated with PSI complex biogenesis and RegA is a modulator for the expression of photosystem genes involved in hydrogen utilization, nitrogen fixation, and carbon fixation (9, 10). A BLASTP analysis using the RefSeq database (release 217) showed 100% identity to proteins with similar potential function from cyanobacteria, suggesting that the MAG has genes associated with photosynthesis and further analysis is needed to conclusively determine the function of these genes. The availability of the bacterial MAG from *Vaucheria* will help in deciphering the role of possible endophytic bacteria in controlling the growth and physiology of algae. Additionally, dissecting the genome of *Pseudomonas* sp. isolated from *Vaucheria bursata* will unravel the intricate interplay between bacteria and algae, providing insights into ecological dynamics and the resilience of aquatic environments.

## Data Availability

MAG was deposited at GenBank under the accession number JBBAGJ000000000. The raw sequencing reads of the MAG are available with the accession number SRX21874967 and were deposited in BioProject PRJNA1020787.
